# Experimental heart failure causes depression-like behavior together with differential regulation of inflammatory and structural genes in the brain

**DOI:** 10.3389/fnbeh.2014.00376

**Published:** 2014-10-31

**Authors:** Anna Frey, Sandy Popp, Antonia Post, Simon Langer, Marc Lehmann, Ulrich Hofmann, Anna-Leena Sirén, Leif Hommers, Angelika Schmitt, Tatyana Strekalova, Georg Ertl, Klaus-Peter Lesch, Stefan Frantz

**Affiliations:** ^1^Medical Clinic and Policlinic I, University Hospital of WürzburgWürzburg, Germany; ^2^Comprehensive Heart Failure Center, University Hospital of WürzburgWürzburg, Germany; ^3^Division of Molecular Psychiatry, Department of Psychiatry, Psychosomatics and Psychotherapy, University Hospital of WürzburgWürzburg, Germany; ^4^Interdisziplinäres Zentrum für Klinische Forschung, University Hospital of WürzburgWürzburg, Germany; ^5^Department of Neurosurgery, University Hospital of WürzburgWürzburg, Germany; ^6^Center of Mental Health, Department of Psychiatry, Psychosomatics, and Psychotherapy, University Hospital of WürzburgWürzburg, Germany; ^7^Department of Neuroscience, School for Mental Health and Neuroscience, Maastricht UniversityMaastricht, Netherlands

**Keywords:** chronic heart failure, myocardial infarction, anxiety, depression, mice

## Abstract

**Background:** Depression and anxiety are common and independent outcome predictors in patients with chronic heart failure (CHF). However, it is unclear whether CHF causes depression. Thus, we investigated whether mice develop anxiety- and depression-like behavior after induction of ischemic CHF by myocardial infarction (MI).

**Methods and Results:** In order to assess depression-like behavior, anhedonia was investigated by repeatedly testing sucrose preference for 8 weeks after coronary artery ligation or sham operation. Mice with large MI and increased left ventricular dimensions on echocardiography (termed CHF mice) showed reduced preference for sucrose, indicating depression-like behavior. 6 weeks after MI, mice were tested for exploratory activity, anxiety-like behavior and cognitive function using the elevated plus maze (EPM), light-dark box (LDB), open field (OF), and object recognition (OR) tests. In the EPM and OF, CHF mice exhibited diminished exploratory behavior and motivation despite similar movement capability. In the OR, CHF mice had reduced preference for novelty and impaired short-term memory. On histology, CHF mice had unaltered overall cerebral morphology. However, analysis of gene expression by RNA-sequencing in prefrontal cortical, hippocampal, and left ventricular tissue revealed changes in genes related to inflammation and cofactors of neuronal signal transduction in CHF mice, with Nr4a1 being dysregulated both in prefrontal cortex and myocardium after MI.

**Conclusions:** After induction of ischemic CHF, mice exhibited anhedonic behavior, decreased exploratory activity and interest in novelty, and cognitive impairment. Thus, ischemic CHF leads to distinct behavioral changes in mice analogous to symptoms observed in humans with CHF and comorbid depression.

## Introduction

Major depression and anxiety disorders are common comorbidities in patients with chronic heart failure (CHF). In the Würzburg heart failure registry (INH registry) approximately 18% of the patients suffered from severe depression (Faller et al., 2009; Angermann et al., 2011), which occurs to happen 3–5 times more often in patients with CHF than in the general population, depending on the severity of CHF symptoms and co-morbidities (Ayuso-Mateos et al., 2001; Rutledge et al., 2006). Major depression is an independent predictor of mortality in CHF patients (Jiang et al., 2004). Remission of depression may improve prognosis of CHF (Jiang et al., 2011). Hospitalization and severe CHF symptoms are recognized as independent predictors of incident depression (Freedland et al., 2003; Lossnitzer et al., 2013). However, the mechanisms of interaction between CHF and the central nervous system are unclear. In humans, this interdependence is complex and influenced by psychosocial and professional impairment (Luttik et al., 2005).

Presently, it is still unclear whether experimental CHF leads to depression-like behavior. A few experimental studies reported behavioral consequences after acute myocardial infarction (MI) (Schoemaker and Smits, 1994; Wann et al., 2007, 2009; Bah et al., 2011). Schoemaker and Smits found increased anxiety-like behavior, diminished interest in a new environment, reduced overall mobility and avoidance of social interaction in rats up to 4 weeks after MI (Schoemaker and Smits, 1994). Wann et al. described depression-like behavior in rats 2 weeks after ligation of the left coronary artery, including anhedonia (i.e., decreased sucrose intake) and behavioral despair (i.e., increased immobility in the forced swim test); both could be reversed by antidepressant treatment (Wann et al., 2007, 2009; Bah et al., 2011). However, these studies lack long term observation and might have been confounded by post-traumatic stress after operation and acute MI.

CHF develops in mice as a function of MI size and time after MI (Hofmann et al., 2012). Functional changes have been reported in brain nuclei of rats with experimental CHF (Hu et al., 2001) and in humans (Almeida et al., 2012). However, the mechanisms and potential systemic consequences, as well as structural or molecular changes in the brain due to CHF are unknown. Moreover, no studies in mice were conducted so far and studies in mice would allow a more feasible genetic manipulation than in rats.

Therefore, we investigated long-term behavioral effects of CHF in mice after MI. We intended to specifically test behavioral and cognitive changes known to be altered in patients with CHF, i.e., depression, motivational deficits, short-term memory impairment. Behavioral tests were selected based on their validity regarding anxiety- and depression-like behavior as well as exploratory behavior and locomotor activity in mice: (1) The sucrose preference test (SPT) as a model for anhedonia, (2) elevated plus maze (EPM), (3) light-dark box (LDB), (4) open field (OF), and (5) object recognition (OR) tests. In these exploration-based tasks, the mouse's innate drive to approach is in conflict with avoidance of a potential threat (Cryan and Holmes, 2005). Mice are expected to avoid aversive areas in the apparatus such as an open, elevated arm (EPM), a brightly lit compartment (LDB) and the center area of a novel, brightly lit open field (OF). Taken together, we established a mouse model suitable to study mechanisms of interdependence between heart failure and its consequences on nervous system functioning. With this experimental setup, we tested the hypothesis whether experimental CHF after myocardial infarction leads to depression- and anxiety-like behavior due to structural and functional brain changes.

## Materials and methods

### Animals

Male C57BL/6N mice (6–9 weeks of age) were obtained from Charles River (Sulzfeld, Germany) and were maintained at ambient temperature (21.5 ± 0.5°C) and humidity (50 ± 5%) under a 14/10 h light/dark cycle (lights on at 07:00 h) with food and water *ad libitum*. Mice were housed in groups of 4–6 per cage and allowed to habituate to the housing facility for at least 1 week before being subjected to MI or sham operation. Animals were housed individually following surgery and the first echocardiography. All animal protocols observed the provisions of the Animal Protection Law according to the Directive of the European Communities Council of 1986 (86/609/EEC), and have been reviewed and approved by the review board of the District Government of Lower Franconia and the University of Würzburg.

### Protocol

**Figure 1:**
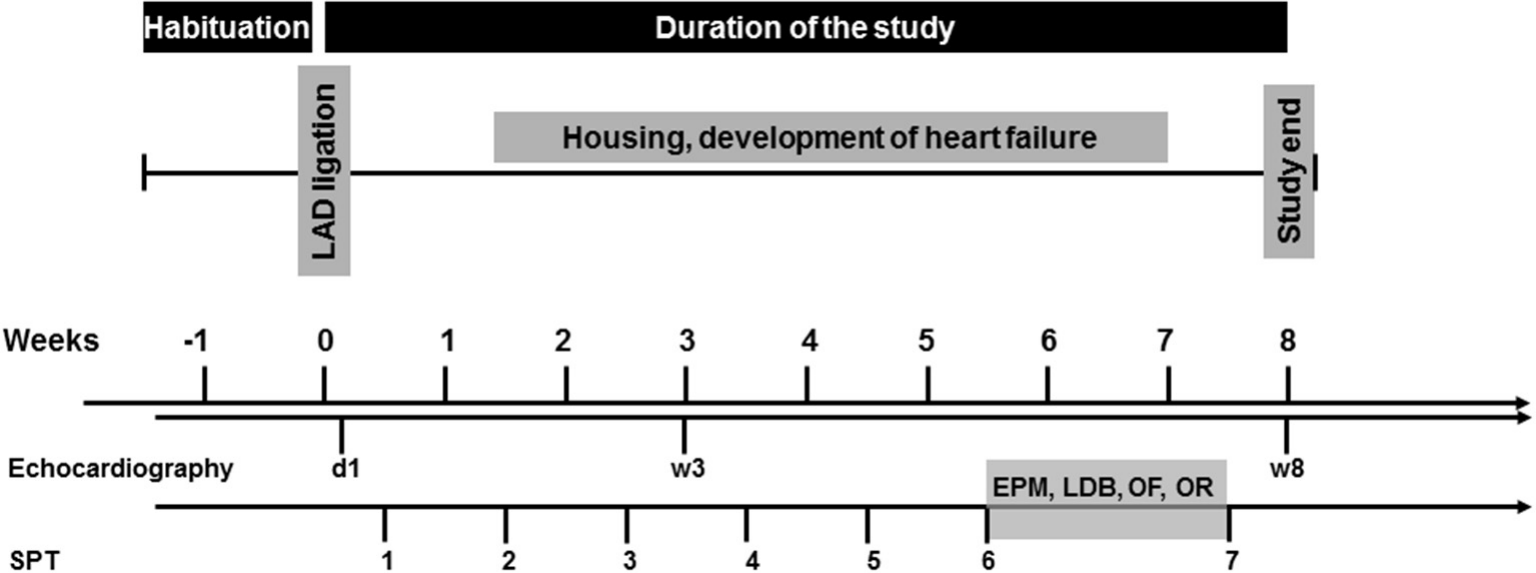
**Experimental design**. LAD, left anterior descending coronary artery; d, day; w, week.

Cardiac operation was initially performed. Starting 4 days after the surgery, mice were tested for anhedonic behavior once per week over a period of 8 weeks using the SPT. 6 weeks after the operation, mice were subjected to a behavioral test battery (in that order: EPM, LDB, OF, OR) to assess exploratory activity, anxiety-like behavior and short-term object recognition memory. The same mice underwent all behavioral tests. All experiments were performed during the light phase between 10:00 and 15:00, with an intertest-interval of 48 h. Echocardiography was performed 1 day, 3 and 8 weeks after surgery. After completion of the experiments, mice were sacrificed and hearts and brains were removed for further analysis. The experimental design is depicted in Figure 1.

### MI and sham operation

MI vs. sham operation was induced in mice according to the established protocol in our department (Hofmann et al., 2012). In brief, mice were anaesthetized with isoflurane (about 2.0 vol.%), intubated and put on a mechanical small animal ventilator. After a left-sided thoracotomy, MI was induced by permanent ligating the proximal portion of the left anterior descending (LAD) coronary artery. Buprenorphin (0.002 mg per mouse) was given i.p. for analgesia perioperatively. In parallel subgroups, thoracotomy was performed to expose the heart but no suture was made to the coronary artery (sham operation).

### Echocardiographic analysis

Echocardiography was performed and evaluated as previously described (Hofmann et al., 2012). Serial ultrasound analyses were performed on a Toshiba Apliosystem with a 15-MHz ultrasound probe under light anesthesia with isoflurane (about 1.5 vol.%) and spontaneous respiration by a single researcher experienced in rodent echocardiography blinded to the type of operation. From two-dimensional short axis imaging, endocardial borders were traced at end-systole and end-diastole utilizing a prototype off-line analysis system (NICE, Toshiba Medical Systems, The Netherlands). Measurements were performed at the mid-papillary muscle level. The end-systolic (smallest) and end-diastolic (largest) cavity areas were determined. Using the end-systolic and -diastolic areas, fractional area changes were calculated [(end-diastolic area—end-systolic area)/end-diastolic area]. From two-dimensionally targeted M-mode tracings, end-diastolic diameter and end-systolic diameter were measured. Fractional shortening was calculated. Only mice with a heart rate greater than 450/min were included in the analysis.

### Sucrose preference test

Mice were given concurrent access to one bottle of 1% (w/v) sucrose solution and one bottle of tap water for 48 h per week. The bottles were weighed after 24 h and their positions switched in order to minimize possible effects of side-preference. Sucrose preference was calculated as the percentage of sucrose solution consumed relative to total liquid intake, with a reduced sucrose preference being an indicator of anhedonic behavior.

### Elevated plus maze

The EPM was made from black perspex (TSE Systems), semipermeable for infrared light, and illuminated by infrared LEDs from below (Post et al., 2011). The apparatus was elevated to a height of 60 cm above floor level and comprised two opposing open arms (30 × 5 × 0.25 cm, 30lx) and two opposing closed arms (30 × 5 × 15 cm, 5lx) extending from a central platform (5 × 5 cm, 15lx). Mice were placed in the center, facing an open arm, and their behavior was recorded for 10 min using the VideoMot2 system (TSE Systems, Bad Homburg, Germany). Behavioral measures included total distance traveled, time spent moving, vertical rears, number of entries, and time spent in each zone of the maze, frequency and duration of grooming bouts and defecation/urination.

### Light-dark box

The LDB comprised a transparent, brightly illuminated'lit' compartment (40 × 40 × 27 cm, 300lx) and a small enclosed “dark” compartment (40 × 20 × 27 cm, 0–5lx) with a central gate (5 × 5 cm) (Bourin and Hascoet, 2003). Mice were placed into the dark compartment and their behavior was recorded for 10 min. Behavioral measures included the latency and time mice spent head-poking into the lit compartment, the latency and time mice spent in the lit compartment and defecation/urination.

### Open field

The OF consisted of a gray opaque PVC box (82 × 82 × 25 cm), divided into a 52 × 52 cm center zone (100lx) and the surrounding periphery (50lx). Mice were placed in the periphery and their behavior was recorded for 10 min using the VideoMot2 system (TSE Systems, Bad Homburg, Germany) (Post et al., 2011). Variables measured included total distance traveled, time spent moving, vertical rears, number of entries, and time spent in the center, frequency, and duration of grooming bouts and defecation/urination.

### Object recognition

The test was performed 24 h after the OF in the same testing arena, i.e., the OF test served as habituation phase. During training (familiarization phase), mice explored two identical objects (A_1_ and A_2_) for 10 min. After a 1 h-retention interval, mice were allowed to explore one familiar (A_2_) and one novel (B) object for 10 min. The novel object location was counterbalanced between groups to avoid potential effects of side-preference. The discrimination index (%) was calculated as ratio of the time mice spent exploring one of the two objects during training (A_1_) or the novel object in the test phase (B) over the total time exploring both objects, i.e., A_1_/(A_1_ + A_2_) × 100 (training) and B/(B + A_2_) × 100 (retention test). Thus, an index >50% indicates novel object preference, <50% familiar object preference, and 50% no preference (Hammond et al., 2004).

### Sample collection, determination of infarct size, ventricular remodeling, and quantification of heart failure

After completion of the experiments, animals were anesthetized with a lethal dose of 0.25% tribromoethanol (Avertin) (0.125 mg/g, i.p.). The brains were removed and snap frozen in isopentane. The organs were weighed and stored in paraffin or liquid nitrogen. The left ventricle was cut into three transverse sections: apex, middle ring and base as previously reported (Hofmann et al., 2012). From the middle ring, 5 μm-sections were stained with picrosirius red. Infarct size (fraction of the infarcted left ventricle) was calculated as the percentage of length of circumference. Sham operated mice had no signs of myocardial infarction. Animals with an infarct size of at least 30% were attributed to the ischemic CHF group. In the correlation analysis between the size of MI and behavioral features, all animals with a quantifiable MI were included. From non-infarcted myocardium α-MHC, β-MHC, ANP-1, SERT, 5HT2A, and 5HT2B mRNA was quantified by real-time PCR. RNA isolation and real-time polymerase chain reaction procedures were performed with commercially available RNA extraction kit and cDNA transcription kit (Quiagen N.V.) and TaqMan probes (Applied biosystems, Foster City) as reported previously (Hofmann et al., 2012). GAPDH was used as the reference gene.

### Serotonin ELISA

Serotonin values in serum and left ventricular myocardium were assessed according to Uscn Serotonin ELISA E92890Mu (Uscn Life Science, China). Protein was extracted out of serum and left myocardial tissue. The total amount of protein in myocardium was measured with Qubit® Protein Assay (Invitrogen™|Life Technologies, USA). Serotonin ELISA was performed according to the protocol of Uscn Serotonin ELISA E92890Mu (Uscn Life Science, China). To further compare the results, the values of ELISA were referred to the amount of serum in ml or the amount of protein in μg.

### Brain histology and immunohistochemistry

Whole mouse brains were cut into 18 μm thick sagittal sections on a cryostat (Leica, Wetzlar, Germany) and kept in −20°C.

For immunohistochemistry, sections were fixed in 4% paraformaldehyde (20 min) and washed with PBS 3 times. For peroxidase labeling, sections were incubated with 3% hydrogen peroxide in methanol for 20 min to quench endogenous peroxidases, permeabilized and blocked with 10% normal serum of host species from which respective secondary antibodies were derived for 1 h at room temperature. Sections were incubated with mouse anti-NeuN (1:500, Millipore, Schwalbach, Germany), mouse anti-MAP2 (1:500, Millipore, Schwalbach, Germany), mouse anti-GFAP (1:1000, Novocastra, Berlin, Germany), rabbit anti-nestin (1:500, BD Pharmingen, Heidelberg, Germany), goat anti-doublecortin (1:500, C-18, Santa Cruz Biotech, Santa Cruz, CA, USA), mouse anti-beta-tubulin III (1:200, Sigma-Aldrich, Taufkirchen, Germany), rabbit anti-Pax6 (1:200, Millipore, Schwalbach, Germany), rat anti mouse MAC-1 (CD11b, 1:50, Serotec, Düsseldorf, Germany), mouse non-phosphorylated neurofilament (anti-SMI32, 1:1000, Covance, Munich, Germany), or rabbit anti cleaved caspase-3 (1:500, New England Biolabs, Frankfurt am Main, Germany) antibodies respectively diluted in 3% normal serum, 0.5% Triton-X in PBS for 24 h at 4°C. After 3 washes with PBS, sections were incubated with biotinylated secondary antibodies for 1 h and the staining was visualized by a peroxidase-labeled avidin-biotin kit (Vector Laboratories, Burlingame, CA, USA). The sections were allowed to dry overnight and mounted using DePeX (Serva, Heidelberg, Germany).

For Nissl staining, sections were immersed for 25 min in a dilute cresyl violet stain (0.01%) in acetate buffer (pH 4.5). After being dehydrated in serial dilutions of ethyl alcohol, sections were mounted using DePeX (Byts et al., 2008).

### RNA sequencing in brain tissue

RNA sequencing analysis was performed in two brain regions (prefrontal cortex, and hippocampus) and left ventricular myocardium. Total RNA was extracted according to the Direct-Zol RNA Miniprep kit (Zymo Research Corporation, Irvine). RNA of 3 CHF mice vs. 6 sham operated mice was pooled and further subjected to RNA sequencing. To fabricate singular pools each probe of the group equally contributed with the final amount of 2 μg RNA per pool with the result that we had 6 different pools to be further evaluated. The RNA sequencing was performed by IGA Technology, Italy (http://www.igatechnology.com/). RNA samples were processed using TruSeq Stranded Total RNA with Ribo-Zero Human/Mouse/Rat from Illumina (Illumina, Inc., CA, USA). Briefly, the total RNA was treated with Ribo-Zero to remove cytoplasmic rRNA, fragmented into small pieces using divalent cations under elevated temperature, cDNA was synthesized by reverse transcription and standard blunt-ending plus add “A” was performed. Then, Illumina TruSeq adapters with indexes were ligated to the ends of the cDNA fragments. After ligation reaction and separation of not ligated adapters, samples were amplified by PCR to selectively enrich those cDNA fragments in the library having adapter molecules at both ends. A pool of the 4 samples was loaded on two lane of Illumina flowcell and clusters created by Illumina cBot. The clusters were sequenced at ultra-high throughput on the Illumina HiSeq2000 (Illumina Inc.). Two lanes in 4-plex were run obtaining about 50 millions of single-reads per sample, 50 bp long. CLC-Bio Genomics Workbench software (CLC Bio, Denmark) was used to calculate gene expression levels based on Mortazavi et al. approach. The sensitivity of RNA-Seq is a function of both molar concentration and transcript length. We therefore quantified transcript levels in reads per kilobase of exon model per million mapped reads (RPKM). The RPKM measure of read density reflects the molar concentration of a transcript in the starting sample by normalizing for RNA length and for the total read number in the measurement. This facilitates transparent comparison of transcript levels both within and between samples. The reference genome used to align the reads was GRCm38 downloaded at the following ftp address: ftp://ftp.ncbi.nlm.nih.gov/genbank/genomes/Eukaryotes/vertebrates_mammals/Mus_musculus/GRCm38.p1/Primary_Assembly/assembled_chromosomes/FASTA/.

Only genes with the number of at least 100 unique gene reads were considered for data analysis. Comparisons within the groups were based on the ratio of RPKM values of each singular gene and were performed logarithmically (Mortazavi et al., 2008). Relevant up- or down-regulation of gene expression was defined as a log2 fold-change (logarithmized ratio of RPKM in CHF over RPKM in sham-operated mice) of ≥0.9 or ≤−0.9, which means ≥1.86 fold decrease or increase, in CHF mice over sham-operated animals.

### Statistical analysis

Statistical analyses were performed using IBM SPSS Statistics 19 (IBM, Armonk, New York) or StatView 5.0 (Abacus Concepts, Berkley, CA, USA). Differences between groups (CHF vs. sham) were analyzed using unpaired *t*-tests. In addition, paired *t*-tests were performed within each group to assess habituation in the object recognition test (training vs. retention test). Data obtained from the sucrose preference test were analyzed via Two-Way mixed ANOVA with group as between-subjects factor and time as repeated measures factor, followed by planned contrasts to further analyse significant main effects of time or time x group interactions. Correlations between MI size and behavioral measures were analyzed using Pearson's correlation (one-tailed); all animals with a quantifiable MI were included in these analyses. A probability of *p* < 0.05 was considered statistically significant. All results are expressed as mean ± s.e.m.

## Results

### Cohort description

13 CHF mice with MI size ≥30% (50.2 ± 2.8%) and 16 mice with sham operation were included in the analysis (Table 1). For correlation analysis, data of all mice with measurable infarct size (*n* = 19; *MI* = 37.7 ± 4.0%) were included.

**Table 1:** **Infarct size, organ and body weights 8 weeks after surgery in CHF vs. sham mice**.

	**CHF after MI**	**Sham**
Infarct size, %	50 ± 2.8	0
RV weight/BW weight, mg/g	1.07 ± 0.05^**^	0.86 ± 0.03
LV weight/BW weight, mg/g	4.87 ± 0.21^**^	3.44 ± 0.09
Lung weight/BW weight, mg/g	6.28 ± 0.33	5.55 ± 0.23
Body weight, g	28.5 ± 0.3	28.4 ± 0.5

Mean ± s.e.m. RV, right ventricle; LV, left ventricle; BW, body weight. (^*^p < 0.05,

** *p < 0.01, CHF vs. Sham)*.

Echocardiography showed upon ligation of the LAD wall motion abnormalities of the left chamber, reduced fraction shortening (*p* < 0.001), and left ventricular chamber dilation (*p* < 0.001) (Table 2) in line with heart failure. No histological and echocardiographic changes typical for MI were observed in the sham group (Supplementary Figure 1). The relative weight of the right ventricle (*p* = 0.002) and the left ventricle (*p* < 0.001) was increased in mice with ischemic CHF (Table 1). Additionally, markers of heart failure were increased (β-MHC [763 ± 32 vs. 27 ± 14 arb. units; *p* = 0.001]; ANP-1 [886 ± 475 vs. 359 ± 556 arb. units; *p* = 0.006]) or decreased (α-MHC [1230 ± 320 vs. 1670 ± 260 arb. units; *p* = 0.0099]) on the RNA-level in mice with myocardial infarction as expected (Van Rooij et al., 2007; Ghosh and Haddad, 2011). Further relevant changes of inflammatory cytokines (e.g., 2-fold increase of TGF-β 1, 3-fold increase of IL10 receptor, 2-fold decrease of IL15) were found in myocardial tissue of CHF mice in RNA-sequencing analysis.

**Table 2:** **Echocardiographic measurements 1 day and 8 weeks after surgery in CHF vs. sham mice**.

	**1 day**		**8 weeks**	
	**CHF after MI**	**Sham**	**CHF after MI**	**Sham**
ESA [mm^2^]	8.87 ± 0.57^**^	5.75 ± 0.72	21.69 ± 2.24^**^	4.96 ± 0.61
EDA [mm^2^]	10.74 ± 0.57^*^	8.39 ± 0.68	24.36 ± 2.18^**^	7.54 ± 0.6
2D FS [%]	18 ± 2^**^	34 ± 3	13 ± 2^**^	36 ± 3
ESD [cm]	0.35 ± 0.02^**^	0.26 ± 0.01	0.57 ± 0.04^**^	0.27 ± 0.01
EDD [cm]	0.40 ± 0.02	0.36 ± 0.01	0.62 ± 0.03^**^	0.36 ± 0.01
FS [%]	11 ± 1^**^	28 ± 2	8 ± 1^**^	25 ± 2
HR [1/min]	488 ± 15	552 ± 15	548 ± 23	578 ± 15

Mean ± s.e.m. EDA, end-diastolic area; ESA, end-systolic area; FS, fractional shortening; 2D, two-dimensional; ESD, end-systolic diameter; EDD, end-diastolic diameter; HR, heart rate. (

* *p < 0.05*,

** *p < 0.01, CHF vs. sham)*.

### Sucrose preference test

#### Total liquid intake

**Figure 2:**
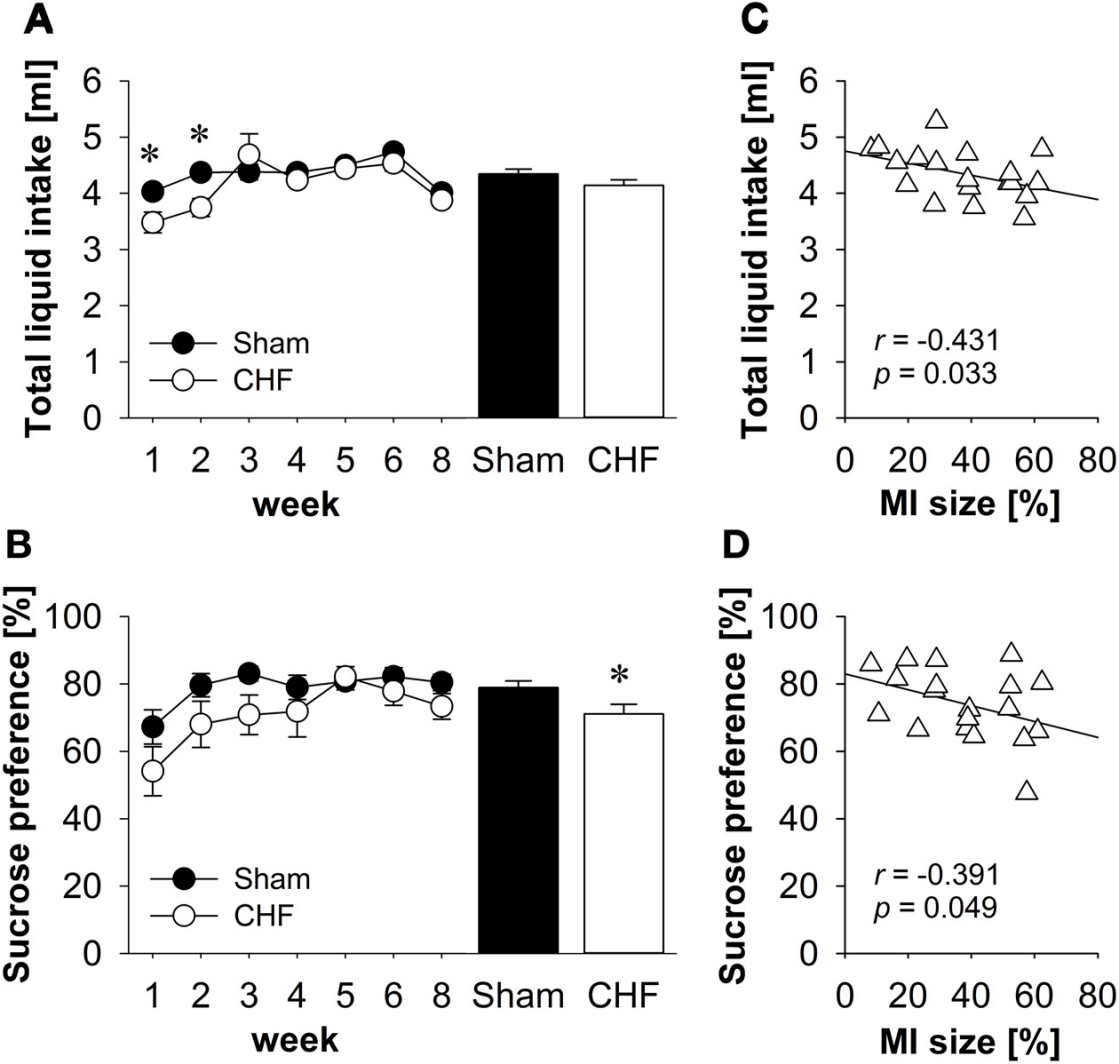
**Sucrose preference test**. **(A)** Total liquid intake was significantly decreased in CHF mice during the first 2 weeks after surgery, whereas no differences were observed between 3 and 8 weeks after the operation. **(B)** The average preference for sucrose was significantly diminished in CHF mice (*n* = 13) compared to sham controls (*n* = 16). Averaged total liquid intake **(C)** and sucrose preference **(D)** were negatively correlated with MI size (*n* = 19). Mean ± s.e.m. ^*^p < 0.05.

Repeated-measures ANOVA found a significant time x group interaction for total liquid intake (*p* = 0.034; Figure 2A). Follow-up comparisons revealed that liquid consumption was significantly decreased in CHF mice during the first 2 weeks post-surgery (*p* < 0.01), whereas no differences between CHF and sham mice were observed between 3 and 8 weeks after the operation.

#### Water intake

Repeated-measures ANOVA indicated that water intake did not change in both groups over the 8-week testing period. However, on average, CHF mice consumed more water than sham controls (*p* = 0.051; Supplementary Table 1).

#### Sucrose intake

Repeated-measures ANOVA revealed that sucrose intake changed significantly in both groups over the 8-week testing period (time effect: *p* < 0.001). Repeated contrasts indicated an increase in sucrose consumption during the first 3 weeks post-surgery and a drop in sucrose intake in the last testing session compared to the preceding one. On average, CHF mice consumed less sucrose than sham controls (*p* = 0.029; Supplementary Table 1).

#### Sucrose preference

Repeated-measures ANOVA found a significant effect of time for sucrose preference (*p* < 0.01; Figure 2B). Follow-up comparisons revealed that sucrose preference was significantly decreased in both groups during the first week post-surgery compared to all other points in time (all *p* < 0.05). Moreover, the average preference for sucrose was significantly diminished in CHF mice compared to sham controls (*p* = 0.029; Supplementary Table 1).

#### Correlation

Pearson's correlation coefficients (Supplementary Table 1) revealed a significant negative relation between MI size and the averaged total liquid intake (*p* = 0.033; Figure 2C), the sucrose intake (*p* = 0.022) and the sucrose preference (*p* = 0.049; Figure 2D).

### Elevated plus maze

**Figure 3:**
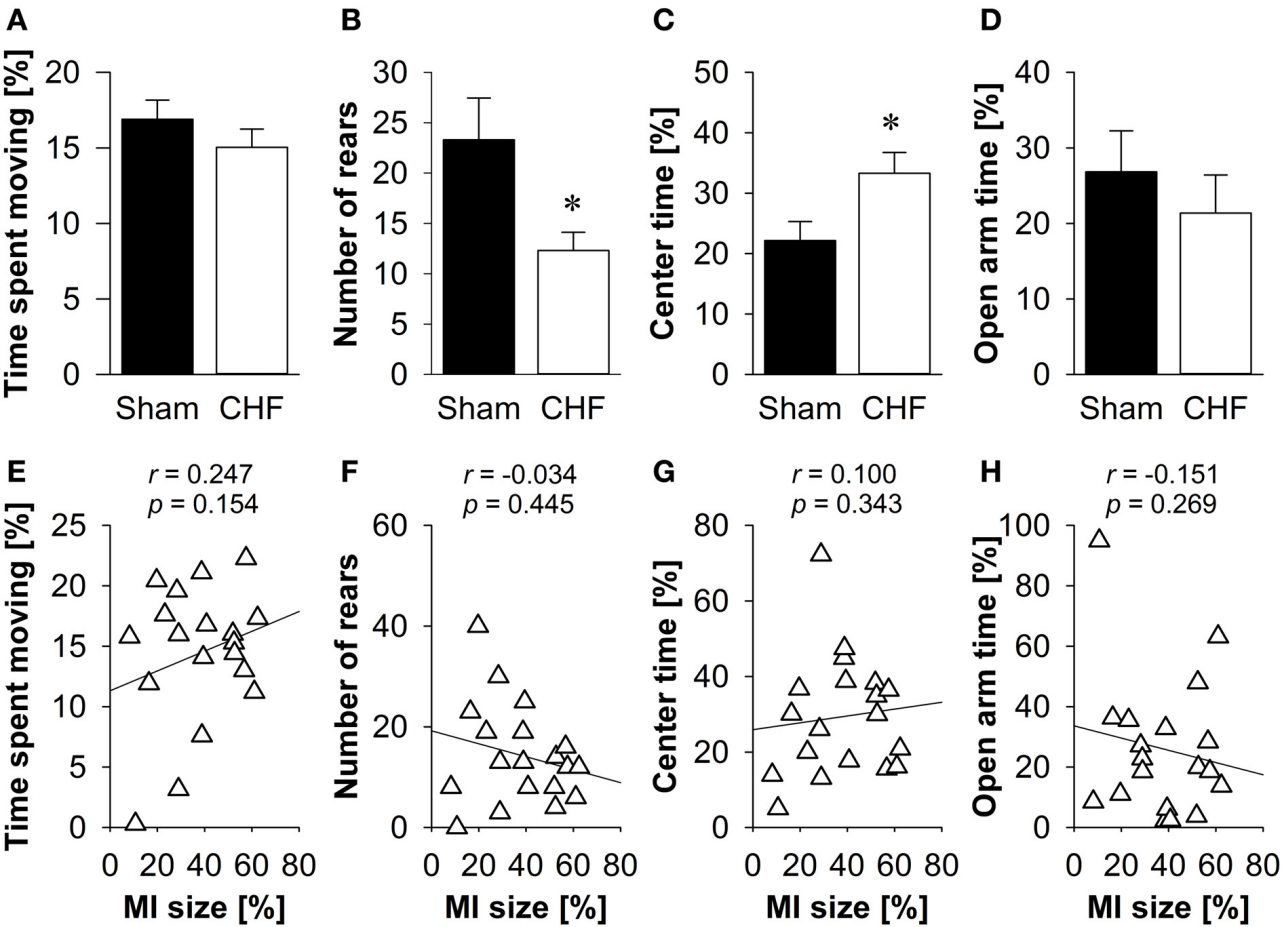
**Elevated plus maze. (A)** Time spent moving did not differ between the groups, although CHF mice (*n* = 13) made significantly less vertical rears **(B)** and spent more time in the center **(C)** than sham controls (*n* = 16). However, time spent on the open arms **(D)** did not differ. **(E–H)** None of the behavioral measures were correlated with MI size (*n* = 19). Mean ± s.e.m. ^*^p < 0.05.

The time spent moving (Figure 3A), the distance traveled and the relative velocity did not differ between CHF and sham mice. However, CHF mice made significantly less vertical rears (*p* = 0.033; Figure 3B) and spent more time in the center of the EPM (*p* = 0.024; Figure 3C) than sham controls. However, there were no significant differences between groups regarding anxiety-related measures, including the number of entries and time spent on the open arms (Figure 3D), as well as defecation/urination (Supplementary Table 2). Pearson's correlation coefficients (Supplementary Table 2) revealed no significant relations between MI size and any of the variables measured in the EPM (Figures 3E–H).

### Light-dark box

**Figure 4:**
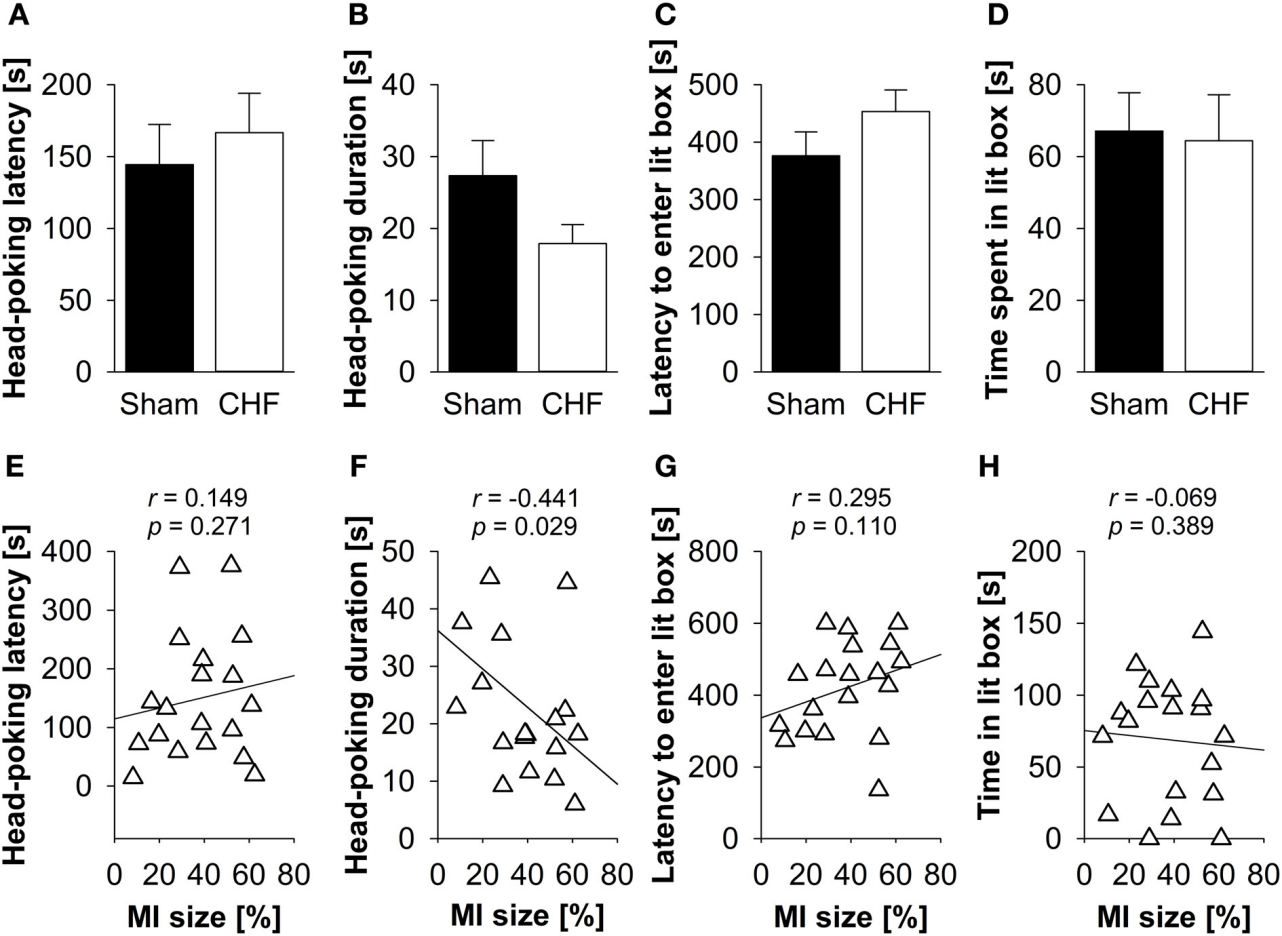
**Light-dark box**. Neither **(A)** head-poking latency and **(B)** head-poking duration nor **(C)** latency and **(D)** time spent in the lit compartment differed between CHF (*n* = 13) and sham mice (*n* = 16). **(E)** Head-poking latency was not correlated with MI size (*n* = 19). However, there was a relation between MI size and **(F)** head-poking duration. **(G)** Latency to enter the lit compartment and **(H)** time spent in the lit compartment were not associated with MI size. Mean ± s.e.m. ^*^p < 0.05.

There was neither a significant difference between CHF and sham mice regarding latency and time mice spent head-poking from the dark into the lit compartment nor latency and time mice spent exploring the lit compartment (Figures, 4A–D), nor in the defecation/urination index (Supplementary Table 3). Pearson's correlation coefficients (Supplementary Table 3) revealed a significant negative relation between MI size and head-poking frequency (*p* = 0.029) as well as head-poking duration (*p* = 0.029; Figure 4F). No correlations were found for MI size and head-poking latency (Figure 4E), latency to enter (Figure 4G) and time spent in the lit compartment (Figure 4H).

### Open field

**Figure 5:**
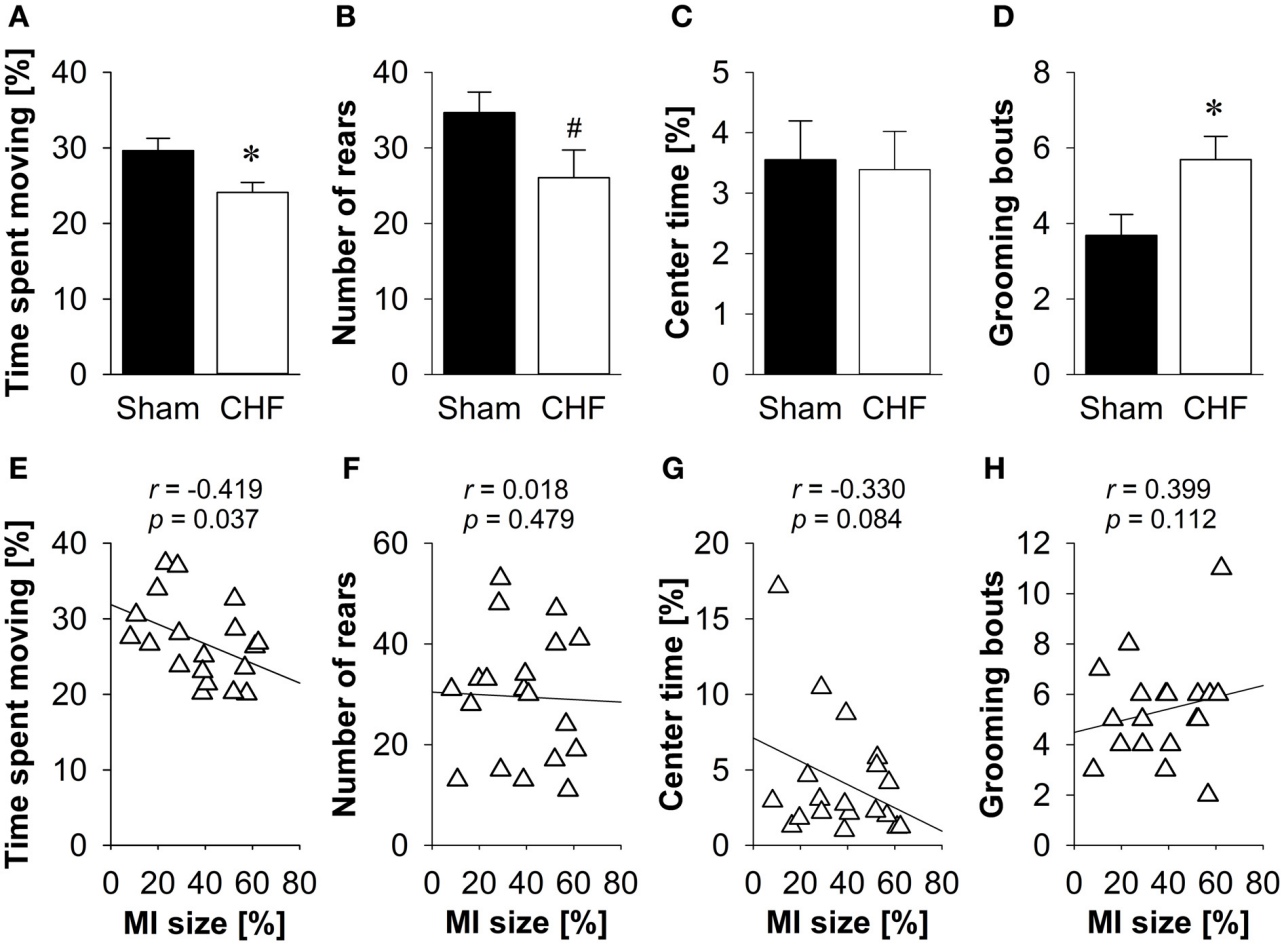
**Open field**. **(A)** Time spent moving and **(B)** number of rears were significantly decreased in CHF mice, although **(C)** time spent in the center did not differ between the groups. **(D)** Number of grooming bouts was significantly increased in CHF mice (*n* = 13) compared to sham controls (*n* = 16). **(E)** Time spent moving was negatively correlated with MI size (*n* = 19), whereas **(F)** number of rears, **(G)** center time and **(H)** grooming frequency were not associated with MI size. Mean ± s.e.m. ^#^*p* < 0.1, ^*^p < 0.05.

CHF mice spent significantly less time moving (*p* = 0.016; Figure 5A) and traveled shorter distances (*p* = 0.021) than sham controls. Moreover, CHF mice displayed a prolonged latency to rear (*p* = 0.009) and tended to make less vertical rears (*p* = 0.064; Figure 5B) as compared to sham controls. However, the relative velocity and anxiety-related measures, including the number of entries and time spent in the center (Figure 5C), as well as defecation/urination (Supplementary Table 4) did not differ between CHF and sham mice. Moreover, CHF mice showed an increased number (*p* = 0.022; Figure 5D) and cumulative duration of grooming bouts (*p* = 0.028). Pearson's correlation coefficients (Supplementary Table 4) revealed a significant negative relation between MI size and time spent moving (*p* = 0.037; Figure 5E). However, no correlation was found for MI size and number of rears, center time and grooming frequency (Figures 5F–H).

### Object recognition

**Figure 6:**
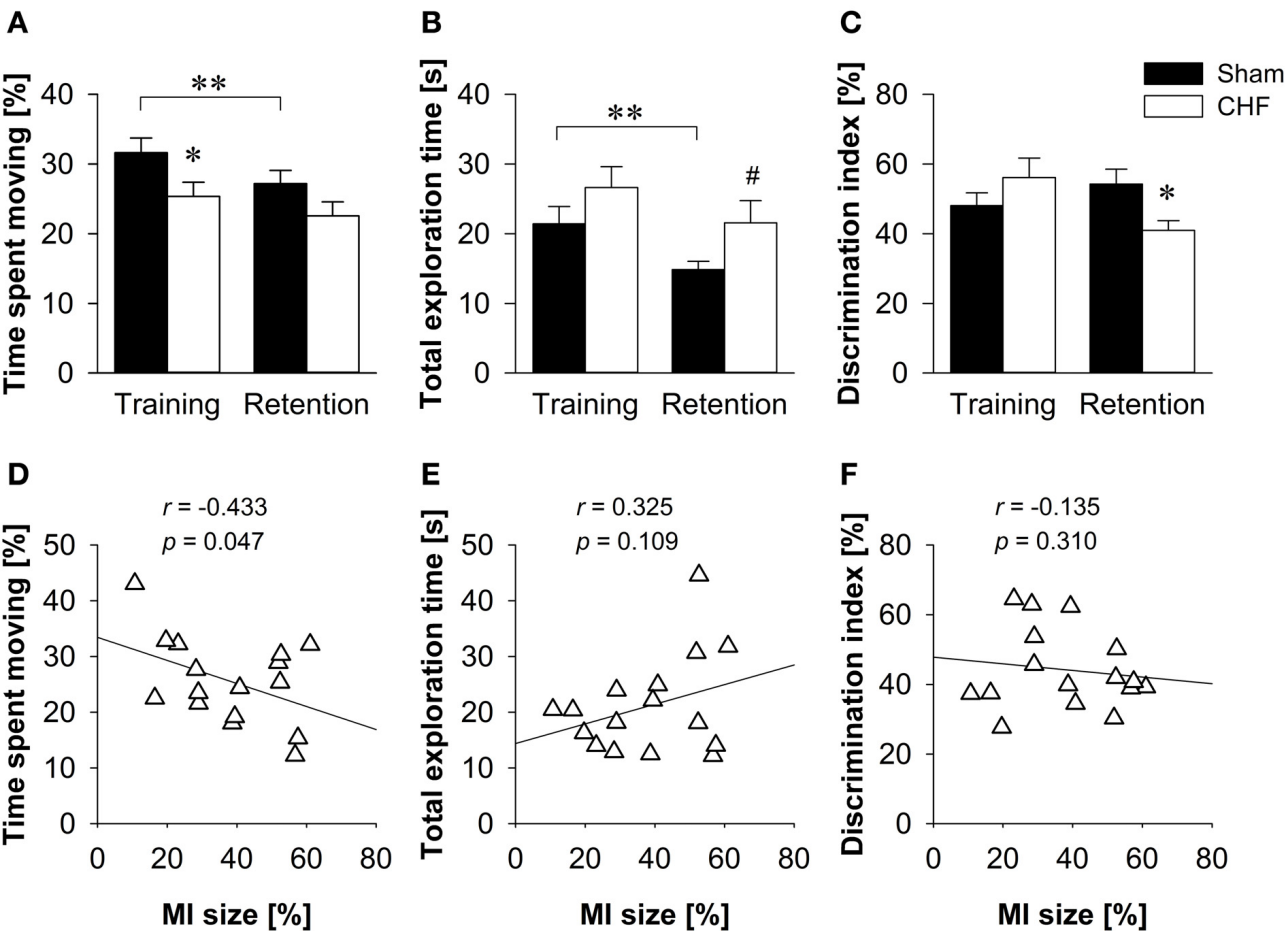
**Object recognition**. **(A)** CHF mice (*n* = 11) spent less time moving than sham controls (*n* = 14) in the training trial, but not in the retention test. **(B)** The total duration of object exploration did not differ between CHF and sham mice during training, but tended to be decreased in sham mice in the retention test. **(C)** The discrimination index for a novel object was significantly decreased in CHF mice in the retention test. **(D)** Time spent moving in the retention test was negatively correlated with MI size (*n* = 16). There was no correlation between MI size and **(E)** total exploration time as well as **(F)** discrimination index. Mean ± s.e.m. ^#^*p* < 0.1, ^*^p < 0.05, ^**^p < 0.01.

CHF mice spent less time moving than sham controls in the training trial (*p* = 0.046), but not in the retention test (Figure 6A). Despite reduced locomotion in CHF mice, the relative velocity did not differ between groups during training. However, CHF mice moved faster than sham controls in the retention test (*p* = 0.035; Supplementary Table 5A). Moreover, the total duration of object exploration did not differ between CHF and sham mice in the training trial, but tended to be decreased in sham mice in the retention test (*p* = 0.071; Figure 6B). During training, both groups showed similar preferences for the two identical objects. In the retention test, however, CHF mice exhibited a significantly decreased discrimination index as compared to sham controls (*p* = 0.022; Figure 6C), reflecting a reduced preference for the novel object. Moreover, paired *t*-tests (Supplementary Table 5B) comparing training and retention test revealed a significant decrease in the distance traveled (*p* < 0.001), time spent moving (*p* < 0.001; Figure 6A), relative velocity (*p* = 0.002), total object exploration time (*p* = 0.008; Figure 6B) and total number of visits (*p* = 0.020) in sham mice. However, none of these measures reached significance in CHF mice, indicating differences between groups in habituating to the objects and the environment. Pearson's correlation coefficients (Supplementary Table 5A) revealed a significant negative correlation between MI size and time spent moving (training: *p* = 0.049; retention test: *p* = 0.047; Figure 6D). However, no correlation was found for MI size and total exploration time as well as the discrimination index in the retention test (Figures 6E,F).

### Serotonin pathway in failing left myocardium

Serum values of serotonin did not differ between CHF and sham-operated animals (227 ± 26 vs. 216 ± 34 ng/ml; *p* = 0.37), but production of serotonin was elevated in the left myocardium of CHF mice compared to sham controls (0.051 ± 0.022 vs. 0.034 ± 0.009 ng/10 μg protein; *p* = 0.042). Additionally, we found increased expression of the serotonin receptor 5HT2B in the failing left myocardial tissue (127 ± 50 vs. 82 ± 42 arb. units; *p* = 0.012) without differences in expression of serotonin transporter (171 ± 106 vs. 110 ± 55 arb. units; *p* = 0.15) and serotonin receptor 5HT2A (296 ± 125 vs. 349 ± 140 arb. units; *p* = 0.44).

### Brain histology and immunohistochemistry

**Figure 7:**
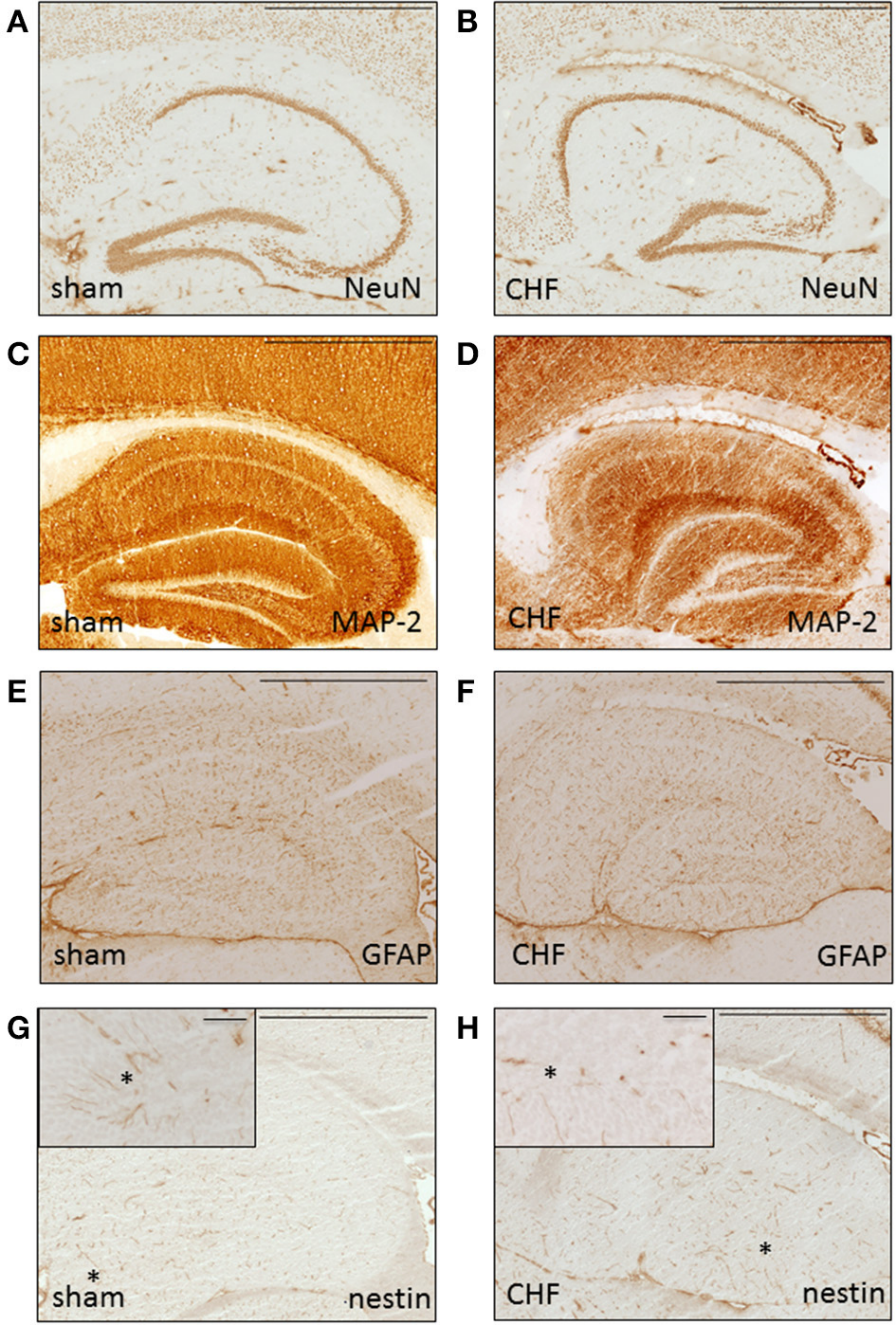
**Histological brain analysis**. Representative immunohistochemical staining without differences for neuronal (**A,B** NeuN, C-D MAP-2), astrocytic (**E,F** GFAP) and neural stem cell (**G,H** nestin) markers in dorsal hippocampus of a sham mouse **(A,C,E,G)** and a CHF mouse **(B,D,F,H)**. Insets in **(G,H)** show high power field of nestin-positive cells (arrow) in the granule cell layer of gyrus dentatus. Scale bar = 100 μm.

Histological analysis of the brains 8 weeks after MI in 5 mice with CHF and 5 sham-operated control mice showed normal general brain morphology in all animals regardless of the extent of the myocardial damage. Immunohistochemical analysis (Figure 7) showed normal expression pattern of markers for neurons (MAP-2, NeuN) and astrocytes (GFAP). Analysis of the brains using markers for neurogenesis (nestin, Pax-6, doublecortin, beta-Tubulin-III) showed no differences between the groups. Staining for markers of inflammation (MAC-1), neurodegeneration (SMI-32), or apoptosis (cleaved caspase 3) were negative in both groups.

### RNA sequencing

**Figure 8:**
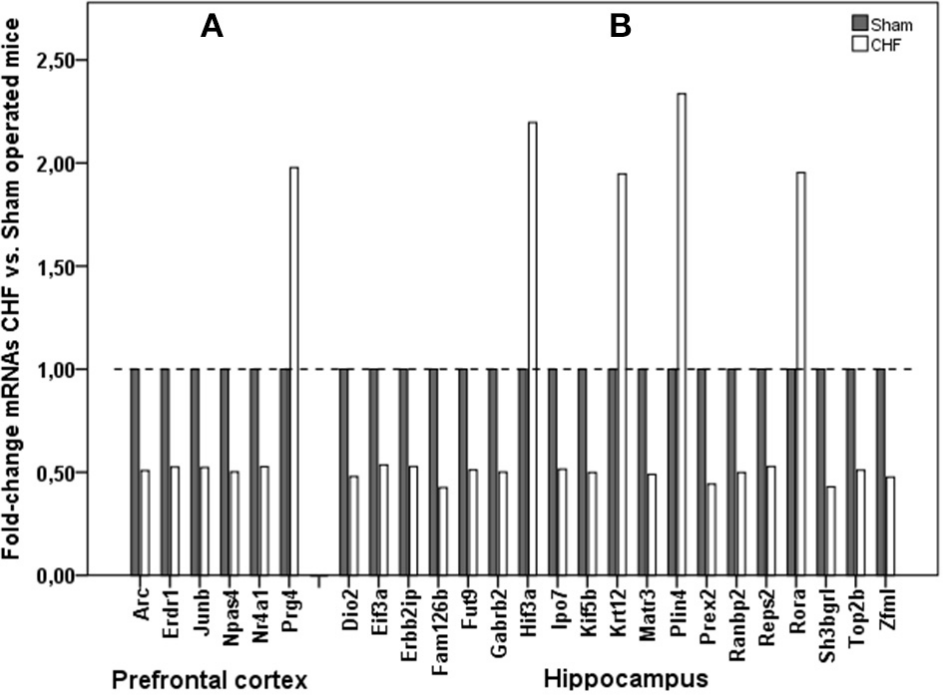
**mRNA sequencing in the brain**. Comparison of mRNA expression in **(A)** prefrontal cortex and **(B)** hippocampus of CHF (*n* = 3) and sham (*n* = 6) mice.

Analysis of gene expression by means of RNA sequencing in prefrontal cortex and hippocampus revealed that 25 confidently detected mRNAs were up- or down-regulated, defined as a log2 fold-change of ≥0.9 or ≤−0.9, which means ≥1.86 fold decrease or increase in CHF mice over sham-operated animals (Figure 8, Supplementary Table 6). A subset of regulated genes of interest was confirmed by RT-PCR to further verify our results of RNA-sequencing analysis. 1182 genes were regulated in the left ventricular myocardium after development of CHF after MI. Nr4a1 was the only gene regulated both in left ventricular tissue (5-fold increase) and in the prefrontal cortex (almost 2-fold decrease) of CHF mice. Further gene regulation of the CHF myocardium is not the main topic of this manuscript and is therefore not presented in detail.

## Discussion

Taken together, our study shows that experimental CHF in mice induced depression-like behavior indicated by a persistent anhedonic-like state, reduced motivation and preference for novelty as well as impaired cognitive abilities.

### Depression and anhedonia in CHF

Anhedonia is a state of decreased ability to experience pleasures or rewards and is a core symptom of clinical depression. In rodents, a decrease in sucrose preference can be regarded as a primary feature of anhedonia, suggesting a depression-like state (Strekalova et al., 2004). Previous studies have shown that acute MI in rats induces anhedonia (Wann et al., 2007, 2009; Bah et al., 2011). Our findings show a persistent anhedonic state in mice with CHF. Lack of differences in body weight between the groups excludes possible metabolic confounders contributing to different sucrose intake. Moreover, sucrose preference was negatively correlated with infarct size, suggesting that severity of the hedonic deficit was associated with the degree of cardiac dysfunction. Despair is also a major symptom of clinical depression. However, we did not perform the forced swim test to assess behavioral despair in anticipation of increased mortality in CHF mice subsequent to the test-induced stressful experience and based on the inherent limitation that the forced swim test was originally designed to test antidepressant drug efficacy.

### Anxiety-like behavior in CHF

To assess anxiety-like behavior and other features of depression-like behavior, such as interest in novelty, exploratory behavior, as well as general locomotor activity, mice were tested in the elevated plus maze, the light-dark box and the open field. The avoidance of the aversive areas and preference of the more protected zones of the respective arenas were similar in both groups in all three tests.

While measures suggestive of anxiety-like behavior were not affected by CHF, CHF mice exhibited both a decreased locomotor activity and a lack of motivation to explore a novel environment. However, EPM, LDB, and OF tests explicitly depend on intact sensory and motor functions. Thus, abnormalities in locomotor activity may confound the results, especially when analyzing conventional spatiotemporal measures such as latency to enter, number of entries and time spent in the aversive area (Cryan and Holmes, 2005). We assessed additional parameters such as rearing, grooming, freezing, head dipping, stretching, defecation and urination to control for these confounders. In the present study, locomotor activity was significantly reduced in CHF mice in the OF, but not in the EPM, whereas vertical rearing activity was significantly affected in both tests. However, the relative velocity did not differ between CHF and sham mice in both EPM and OF, suggesting that CHF did not cause grave physical disabilities. The analysis of vegetative behaviors, as measured by defecation and urination (a conventional emotionality index) in the EPM, LDB, and OF, did not reveal a significant difference between CHF mice and sham controls. Taken together, our data indicate that the reduced exploratory behavior observed in CHF mice was most likely due to a lack of motivation and reduced preference for novelty rather than an anxiety-induced suppression of exploration.

Grooming behavior is an established marker of stress in rodents, including stress-coping and de-arousal (Kalueff and Tuohimaa, 2005). However, rodent's self-grooming can be increased by both stress and comfort conditions (Kalueff and Tuohimaa, 2004). Since exposure to a novel environment induces mild to moderate levels of stress, the grooming behavior observed in our tests can be considered as stress-evoked “displacement” grooming, which is different from low-stress grooming and characterized by frequent and rapid short bursts (Kalueff and Tuohimaa, 2005). Analysis of grooming behavior revealed a significant increase in the number and duration of grooming bouts in CHF mice in the OF, but not in the EPM, suggesting that CHF induced deficits in stress-coping depending on the type of test.

### Cognitive impairment in CHF

To assess non-spatial learning and memory, mice were tested in the OR test, which is based on the spontaneous tendency of rodents to explore a novel object over a familiar one (Ennaceur and Delacour, 1988). Using a 1 h retention interval between training and testing phase, we could show that CHF mice display less habituation to the testing environment and a significantly decreased ability to discriminate between a novel and a familiar object. Thus, CHF induced deficits in short-term recognition memory, which is found in patients with depressive disorder (Kindermann et al., 2012).

### Mechanistic considerations

Elevation of serotonin, being associated with depression, platelet activation and increased cardiovascular risk (Williams, 2012), was found in myocardial tissue, accompanied by increased expression of 5HT2B (one of serotonin receptors), which suggests serotonin system to be involved in remodeling processes during development of CHF. However, other pathways than serotonin system could also be of mechanistic importance to understand the interrelation between the brain and the failing heart: Several brain regions are activated in rats after MI and in CHF patients as for example the hypothalamic paraventricular nucleus (Han et al., 2010; Rana et al., 2010; Almeida et al., 2012). Visual examination of brain MRI can detect damage in patients with CHF in areas regulating depression and executive function, including the right hippocampus and left frontal cortex (Pan et al., 2013). Even though we could not find general structural changes in brains of mice after MI, we could detect differences of RNA expression of several hippocampal and prefrontal cortical genes—both brain regions, whose structural and functional alterations are associated with an increased risk for developing major depressive disorder (Spinelli et al., 2013).

#### Gene expression in the hippocampus

Hypoxia-inducible factors (HIFs) are key mediators of cellular adaptation to hypoxia, but also respond to non-hypoxic stimuli. It is thought that factors containing the alpha-3 subunit are negative regulators of hypoxia-inducible gene expression (Makino et al., 2001). Transcriptional up-regulation of HIF-3alpha as found in CHF mice in hippocampus may therefore represent a typical response to *in vivo* hypoxia, hypoglycaemia, and glucoprivation in the brain after MI and CHF development (Heidbreder et al., 2007).

The nuclear receptor Retinoid-related orphan receptor-alpha (RORA), which is up-regulated in hippocampus of CHF mice, exerts a bi-directional regulation of astrocytes, being neuro-protective and anti-inflammatory, a pluripotent molecular player in constitutive and adaptive astrocyte physiology as adaptation after CHF development (Journiac et al., 2009). Additionally, several genetic studies have found an association between variations of RORA and pro-depressive behavior in humans (Utge et al., 2010), which fits to RORA up-regulation in mice with pro-depressive behavior due to CHF.

Furthermore, there seem to be changes in axonal vesicular transport with subsequent maintenance of presynaptic function and regulation of synaptic plasticity and synaptic transmission in hippocampus after CHF induction as far as we found down-regulation in gene expression of Kif5b, responsible for axonal vesicular transport, and Gabrb2, responsible for regulation of synaptic transmission (Ma et al., 2009). Corresponding to our findings Gabrb2 was found to be decreased in the brain in mood disorders (Zhao et al., 2012).

#### Gene expression in the prefrontal cortex

Furthermore, gene expression in the prefrontal cortex revealed a few regulated genes after MI. We found diminished expression of Npas4, Arc, Junb, and Nr4a1 in CHF. Npas4 has been implicated in the formation and maintenance of inhibitory synapses (Shamloo et al., 2006) and it is transcribed in response to excitatory synaptic activity (Lin et al., 2008). Actual findings suggest that Npas4 participates in the regulation of the balance between neuronal excitation and inhibition by contributing to the maintenance of the inhibitory pathways (Coutellier et al., 2012). Relative hippocampal Npas4 expression was significantly lower in a rat depression model compared to controls (Zhang et al., 2014).

Activity-regulated cytoskeleton-associated protein (Arc, also known as Arg3.1) is a plasticity protein which is believed to play a critical role in learning and memory-related molecular processes (McIntyre et al., 2005). Arc is widely considered to be an important protein in neurobiology because of its activity regulation, localization, and utility as a marker for plastic changes in the brain (Kang et al., 2008). CHF is known to be associated not only with depression but also with cognitive impairment in humans (Angermann et al., 2012; Huijts et al., 2013), thus Arc could be a potential link between CHF and cognition.

Junb as a protooncogen has additional function in behavioral changes and was found to be up-regulated in depression (Orsetti et al., 2009). We found decreased Junb levels in CHF mice with depression-like behavior, with the result that function of Junb cannot be finally established in depression.

Nr4a1 (also, so-called NGFIB) protein is involved in cell cycle mediation, inflammation and apoptosis and is expressed in macrophages and plays a key role in mediating inflammatory responses (Pei et al., 2006). Nr4a1 is the only gene which was regulated both in the brain and in the heart: almost 2-fold decreased in the prefrontal cortex and 5-fold increased in left ventricular myocardium of mice with CHF, which is not surprising as far as macrophages are well known to play a relevant role in remodeling after myocardial infarction (Hamid et al., 2009; Ismahil et al., 2013).

In summary, we can conclude that we did not find histological, structural changes in the brain of CHF mice, but we found relevant changes in the expression of genes responsible for axonal vesicle transport (Kif5b), signal transduction (Arc, Gabrb2), limitation of inflammation (RORA; Nr4a1) and of hypoxic brain damage (Hif3a). Besides, the actual literature describes some of the genes (RORA, Gabrb2, Npas4, and Junb) being associated with depression-like behavior. Nr4a1 significantly regulated in both brain and heart tissue after induction of ischemic CHF could be a potential link and reveals the central role of inflammation in the interrelation of the brain and the failing heart.

### Clinical perspective

CHF in mice leads to depression- like behavior suggesting that this interrelation is independent of human psychosocial stress. The SADHART-CHF trial found that treatment with sertraline compared with placebo did not provide greater reduction in depression or improved cardiovascular status among patients with HF and depression. However, patients were only treated with sertraline for 12 weeks in this study, which might be a too short time (O'connor et al., 2010). Further studies are on their way like the MOOD-HF trial which addresses this question (Angermann et al., 2007). The CHF mouse model may be used for further mechanistic studies and developing therapeutic concepts beyond antidepressants.

## Conclusion

This study shows that experimental CHF in mice induces an anhedonic-like state, which persists 8 weeks after the initial insult. The hedonic deficit was accompanied by other features of depression-like behavior, such as decreased exploratory activity, lessened interest in novelty and impaired stress-coping. The behavioral changes in mice are comparable to symptoms observed in humans with heart failure and comorbid depression. On the molecular level we find evidence for changes in cerebral gene profile related to inflammation and signal transduction. Thus, the present model of behavioral evaluation in mice with CHF after MI gives us new pathophysiologic insights of heart-brain interaction during the process of heart failure development.

## Funding sources

This work was supported by grants from the Bundesministerium für Bildung und Forschung (BMBF01 EO1004) through the Comprehensive Hear Failure Center (Anna Frey, *S*andy Popp, Georg *Er*tl, Klaus-Peter Lesch, Stefan *Fr*antz) and the Deutsche Forschungsgemeinschaft (DFG) through SFB 688 (Georg *Er*tl, Stefan *Fr*antz) and SFB 581, B9 (Klaus-Peter Lesch).

## Author contributions

We hereby confirm that all authors made substantial contributions to the manuscript since they were involved in all steps between conceptualization, design, conduct of the study, analysis and interpretation of data, and drafting of the article. Finally, all authors critically revised its content and approved the submitted version.

### Conflict of interest statement

The authors declare that the research was conducted in the absence of any commercial or financial relationships that could be construed as a potential conflict of interest.
