# Supplementary material for: Experimental heart failure causes depression-like behavior together with differential regulation of inflammatory and structural genes in the brain
Source: Front Behav Neurosci. 2014 Oct 31;8:376. doi: 10.3389/fnbeh.2014.00376 (PMC4215623; doi:10.3389/fnbeh.2014.00376)

***Supplementary Material***

**Chronic heart failure and depression: anhedonia, motivation deficits, and functional brain changes in mice with myocardial infarction**

**Short title: Depression in mice with chronic heart failure**

**Anna Frey^1,3*^, MD, Sandy Popp^2,3*^, Antonia Post^2^, Simon Langer^1,3^, Marc Lehmann^1,3^, Ulrich Hofmann^1,5^, Anna-Leena Sirén^4^, MD, Leif Hommers^2,5^, MD, PhD, Angelika Schmitt^2^, PhD, Tatyana Strekalova^6^, PhD, Georg Ertl^1,3^, MD, Klaus-Peter Lesch^2,3,6^, MD, Stefan Frantz^1,3^, MD**

^1^Medical Clinic and Policlinic I, University Hospital of Würzburg, Würzburg, Germany

^2^Division of Molecular Psychiatry, Department of Psychiatry, Psychosomatics and Psychotherapy, University Hospital of Würzburg, Würzburg, Germany

^3^Comprehensive Heart Failure Center, University Hospital of Würzburg, Würzburg, Germany

^4^Department of Neurosurgery, University Hospital of Würzburg, Würzburg, Germany

^5^Interdisziplinäres Zentrum für Klinische Forschung, University Hospital of Würzburg, Würzburg, Germany

^6^Department of Neuroscience, School for Mental Health and Neuroscience, Maastricht University, Maastricht, Netherlands

*** Correspondence:** Anna Frey, M.D., Universitätsklinikum Würzburg, Deutsches Zentrum für Herzinsuffizienz (DZHI),Comprehensive Heart Failure Center, Straubmühlweg 2a, D-97078 Würzburg, Germany, E-Mail: [frey_a@ukw.de](file:///D:\Documents\My%20Dropbox\Depressionsstudie%20AG%20Frantz\C57BL6\Paper\Paper%20-%20Version%20Frontiers%20Neuroscience\frey_a@ukw.de)

1. **Supplementary Figures and Tables**

## Suplementary Tables

**Supplementary Table 1 Sucrose Preference Test.**

|  | Mean±SEM | | ANOVA | | Correlation | |
| --- | --- | --- | --- | --- | --- | --- |
|  | **CHF** | **Sham** | ***F*** | ***p*** | ***r*** | ***p*** |
| Sucrose intake [ml] | 2.97±0.15 | 3.45±0.14 | 5.32 | **0.029*** | -0.468 | **0.022*** |
| Water intake [ml] | 1.17±0.12 | 0.89±0.08 | 4.15 | 0.051 | 0.264 | 0.138 |
| Total liquid intake [ml] | 4.14±0.10 | 4.34±0.09 | 2.32 | 0.139 | -0.431 | **0.033*** |
| Sucrose preference [%] | 71.17±2.84 | 78.92±1.99 | 5.29 | **0.029*** | -0.391 | **0.049*** |

Mean±SEM. Average values for CHF (*n*=13) and sham mice (*n*=16) over a testing period of 8 weeks. Pearson’s correlation for all MI-sizes (*n*=19). (**p*<0.05)

**Supplementary Table 2 Elevated Plus Maze.**

|  | Mean±SEM | | *t*-Test | | Correlation | |
| --- | --- | --- | --- | --- | --- | --- |
|  | **CHF** | **Sham** | ***T*** | ***p*** | ***r*** | ***p*** |
| Distance traveled [cm] | 899.51±79.63 | 1019.74±86.62 | 1.00 | 0.325 | 0.208 | 0.196 |
| Time spent moving [%] | 15.04±1.21 | 16.90±1.27 | 1.04 | 0.308 | 0.247 | 0.154 |
| Relative velocity [cm/s] | 9.86±0.17 | 9.97±0.24 | 0.35 | 0.727 | 0.176 | 0.235 |
| Entries OA | 13.77±2.39 | 12.06±2.53 | -0.48 | 0.634 | 0.308 | 0.100 |
| Entries CA | 18.62±2.11 | 19.81±1.70 | 0.45 | 0.658 | 0.187 | 0.222 |
| Time OA [%] | 21.37±5.05 | 26.83±5.44 | 0.72 | 0.477 | -0.151 | 0.269 |
| Time CA [%] | 45.30±5.30 | 51.00±4.95 | 0.78 | 0.440 | 0.084 | 0.366 |
| Time C [%] | 33.33±3.41 | 22.17±3.16 | -2.39 | **0.024*** | 0.100 | 0.343 |
| Distance OA [cm] | 75.20±24.69 | 85.07±24.33 | 0.28 | 0.780 | 0.174 | 0.239 |
| Distance CA [cm] | 654.34±68.31 | 748.68±85.10 | 0.84 | 0.410 | 0.142 | 0.281 |
| Distance C [cm] | 169.97±17.39 | 185.99±14.35 | 0.72 | 0.480 | 0.273 | 0.129 |
| Latency OA/CA [s] | 65.99±19.70 | 33.23±11.85 | -1.43 | 0.169 | -0.195 | 0.212 |
| Number of rears | 12.31±1.82 | 23.31±4.15 | 2.25 | **0.033*** | -0.034 | 0.445 |
| Grooming bouts | 3.77±0.56 | 4.44±0.66 | 0.75 | 0.458 | 0.200 | 0.206 |
| Defecation/Urination | 2.38±0.76 | 3.25±0.77 | 0.82 | 0.422 | -0.092 | 0.353 |

Mean±SEM. CHF (*n*=13); Sham (*n*=16). Pearson’s correlation for all MI-sizes (*n*=19). OA, open arms; CA, closed arms; C, center. (**p*<0.05)

**Supplementary Table 3 Light-Dark Box.**

|  | Mean±SEM | | *t*-Test | | Correlation | |
| --- | --- | --- | --- | --- | --- | --- |
|  | **CHF** | **Sham** | ***T*** | ***p*** | ***r*** | ***p*** |
| Number of head-pokes | 8.77±0.91 | 11.88±1.66 | 1.54 | 0.135 | -0.442 | **0.029*** |
| Duration of head-poking [s] | 17.89±2.63 | 27.34±4.91 | 1.59 | 0.124 | -0.441 | **0.029*** |
| Latency to first head-poke [s] | 166.63±27.47 | 144.42±27.94 | -0.56 | 0.580 | 0.149 | 0.271 |
| Entries into lit box | 2.54±0.51 | 3.75±0.67 | 1.39 | 0.177 | -0.193 | 0.214 |
| Time spent in lit box [s] | 64.43±12.81 | 67.10±10.69 | 0.16 | 0.873 | -0.069 | 0.389 |
| Latency to enter lit box [s] | 453.46±37.35 | 376.40±41.40 | -1.35 | 0.188 | 0.295 | 0.110 |
| Defecation/Urination | 1.31±0.46 | 2.50±0.62 | 1.55 | 0.134 | -0.227 | 0.175 |

Mean ± SEM. CHF (*n*=13); Sham (*n*=16). Pearson’s correlation for all MI-sizes (*n*=19). (**p*<0.05)

**Supplementary Table 4 Open Field.**

|  | Mean±SEM | | *t*-Test | | Correlation | |
| --- | --- | --- | --- | --- | --- | --- |
|  | **CHF** | **Sham** | ***T*** | ***p*** | ***r*** | ***p*** |
| Distance traveled [cm] | 2480.36±159.01 | 3132.07±202.64 | 2.44 | **0.021*** | -0.326 | 0.086 |
| Time spent moving [%] | 24.11±1.32 | 29.64±1.62 | 2.56 | **0.016*** | -0.419 | **0.037*** |
| Relative velocity [cm/s] | 17.05±0.41 | 17.51±0.34 | 0.86 | 0.397 | 0.155 | 0.264 |
| Center time [%] | 3.39±0.63 | 3.55±0.65 | 0.18 | 0.863 | -0.330 | 0.084 |
| Center distance [cm] | 266.66±47.38 | 343.99±64.44 | 0.93 | 0.362 | -0.326 | 0.087 |
| Center entries | 9.00±1.62 | 10.50±1.64 | 0.64 | 0.526 | -0.167 | 0.247 |
| Center latency [s] | 188.20±36.78 | 140.06±45.59 | -0.80 | 0.433 | 0.353 | 0.069 |
| Number of rears | 26.08±3.66 | 34.69±2.70 | 1.94 | 0.064 | 0.018 | 0.479 |
| Time spent rearing [s] | 21.22±2.83 | 26.30±2.24 | 1.43 | 0.165 | -0.108 | 0.376 |
| Latency to first rear [s] | 221.30±28.66 | 124.42±20.84 | -2.80 | **0.009**** | 0.209 | 0.268 |
| Grooming bouts | 5.69±0.61 | 3.69±0.55 | -2.43 | **0.022*** | 0.399 | 0.112 |
| Time spent grooming [s] | 18.23±1.93 | 12.52±1.56 | -2.33 | **0.028*** | 0.277 | 0.205 |
| Latency to groom [s] | 197.05±23.71 | 169.03±17.46 | -0.97 | 0.340 | 0.204 | 0.274 |
| Defecation/Urination | 2.00±0.57 | 2.00±0.58 | 0.00 | 1.000 | 0.125 | 0.305 |

Mean ± SEM. CHF (*n*=13); Sham (*n*=16). Pearson’s correlation for all MI-sizes (*n*=19). (**p*<0.05, ***p*<0.01)

**Supplementary Table 5A Object Recognition.**

|  | Mean±SEM | | *t*-Test | | Correlation | |
| --- | --- | --- | --- | --- | --- | --- |
|  | **CHF** | **Sham** | ***T*** | ***p*** | ***r*** | ***p*** |
| **Training phase** |  |  |  |  |  |  |
| Distance traveled [cm] | 2736.47±178.14 | 3134.07±189.41 | 1.50 | 0.148 | -0.380 | 0.073 |
| Time spent moving [%] | 25.36±2.03 | 31.64±2.11 | 2.10 | **0.046*** | -0.428 | **0.049*** |
| Relative velocity [cm/s] | 18.25±0.52 | 16.82±0.80 | -1.49 | 0.150 | 0.302 | 0.128 |
| Total exploration time [s] | 26.64±2.99 | 21.42±2.48 | -1.36 | 0.188 | 0.067 | 0.402 |
| Total number of visits [n] | 18.18±2.75 | 19.07±3.03 | 0.21 | 0.834 | 0.075 | 0.391 |
| Discrimination index [%] | 56.14±5.58 | 48.05±3.69 | -1.25 | 0.223 | 0.403 | 0.061 |
| **Retention test** |  |  |  |  |  |  |
| Distance traveled [cm] | 2406.17±198.41 | 2528.37±169.43 | 0.47 | 0.642 | -0.285 | 0.142 |
| Time spent moving [%] | 22.56±2.00 | 27.18±1.92 | 1.65 | 0.113 | -0.433 | **0.047*** |
| Relative velocity [cm/s] | 18.04±0.60 | 15.80±0.80 | -2.25 | **0.035*** | 0.334 | 0.103 |
| Total exploration time [s] | 21.57±3.19 | 14.86±1.18 | -1.97 | 0.071 | 0.325 | 0.109 |
| Total number of visits [n] | 15.73±2.16 | 12.07±2.00 | -1.24 | 0.229 | 0.294 | 0.134 |
| Discrimination index [%] | 40.94±2.80 | 54.28±4.26 | 2.46 | **0.022*** | -0.135 | 0.310 |

Mean ± SEM. CHF (*n*=11); Sham (*n*=14). Pearson’s correlation for all MI-sizes (*n*=16). (**p*<0.05)

**Supplementary Table 5B Object Recognition.**

|  | CHF | | Sham | |  | |
| --- | --- | --- | --- | --- | --- | --- |
| **Paired *t*-test** | ***T*** | ***p*** | ***T*** | ***p*** |  |  |
| Distance traveled [cm] | 1.77 | 0.106 | 6.14 | **<0.001**** |  |  |
| Time spent moving [%] | 1.74 | 0.112 | 5.89 | **<0.001**** |  |  |
| Relative velocity [cm/s] | 0.64 | 0.537 | 3.94 | **0.002**** |  |  |
| Total exploration time [s] | 1.91 | 0.085 | 3.12 | **0.008**** |  |  |
| Total number of visits [n] | 1.53 | 0.158 | 2.66 | **0.020*** |  |  |

Paired *t*-test (training vs. retention test) for CHF (*n*=11) and Sham (*n*=14) mice. (**p*<0.05, ***p*<0.01)

**Supplementary Table 6 Results of mRNA gene sequencing**

| **Brain region** | **Gene**  **name** | **Unique gene reads** | | **RPKM** | | **Fold change**  **CHF/Sham** | **Log2 fold change**  **Log2 (CHF/Sham)** |
| --- | --- | --- | --- | --- | --- | --- | --- |
|  |  | **CHF** | **Sham** | **CHF** | **Sham** |  |  |
| HIPP | Fam126b | 177 | 378 | 0.101 | 0.238 | 0.43 | - 1.23 |
| HIPP | Sh3bgrl | 235 | 488 | 0.125 | 0.291 | 0.43 | - 1.22 |
| HIPP | Prex2 | 143 | 296 | 0.018 | 0.040 | 0.44 | - 1.18 |
| HIPP | Zfml | 128 | 245 | 0.065 | 0.136 | 0.48 | - 1.07 |
| HIPP | Dio2 | 181 | 340 | 0.473 | 0.987 | 0.48 | - 1.06 |
| HIPP | Matr3 | 631 | 1145 | 0.750 | 1.532 | 0.49 | - 1.03 |
| HIPP | Kif5b | 413 | 742 | 0.372 | 0.747 | 0.50 | - 1.00 |
| HIPP | Ranbp2 | 154 | 273 | 0.116 | 0.231 | 0.50 | - 1.00 |
| HIPP | Gabrb2 | 401 | 716 | 0.068 | 0.135 | 0.50 | - 1.00 |
| HIPP | Top2b | 184 | 322 | 0.100 | 0.196 | 0.51 | - 0.97 |
| HIPP | Fut9 | 131 | 229 | 0.025 | 0.048 | 0.51 | - 0.97 |
| HIPP | Ipo7 | 149 | 257 | 0.144 | 0.280 | 0.52 | - 0.96 |
| HIPP | Erbb2ip | 161 | 274 | 0.059 | 0.112 | 0.53 | - 0.92 |
| HIPP | Reps2 | 595 | 1012 | 0.094 | 0.178 | 0.53 | - 0.92 |
| HIPP | Eif3a | 605 | 1021 | 0.768 | 1.433 | 0.54 | - 0.90 |
| HIPP | Krt12 | 313 | 139 | 2.336 | 1.200 | 1.95 | 0.96 |
| HIPP | Rora | 356 | 166 | 0.019 | 0.010 | 1.95 | 0.97 |
| HIPP | Hif3a | 317 | 128 | 0.360 | 0.164 | 2.20 | 1.14 |
| HIPP | Plin4 | 326 | 124 | 1.256 | 0.538 | 2.34 | 1.22 |
| PFC | Npas4 | 447 | 1076 | 3.941 | 7.856 | 0.50 | - 1.00 |
| PFC | Arc | 2596 | 6161 | 36.856 | 72.439 | 0.51 | - 0.98 |
| PFC | Junb | 902 | 2080 | 24.276 | 46.360 | 0.52 | - 0.93 |
| PFC | Erdr1 | 151 | 327 | 1.456 | 2.769 | 0.53 | - 0.92 |
| PFC | Nr4a1 | 2289 | 5246 | 14.258 | 27.062 | 0.53 | - 0.92 |
| PFC | Prg4 | 279 | 171 | 0.842 | 0.426 | 1.98 | 0.98 |

**1.2. Supplementary Figures**

**Supplementary Figure 1 Picrosirius red staining**

(A) CHF mouse, (B) mouse with sham operation.

1.
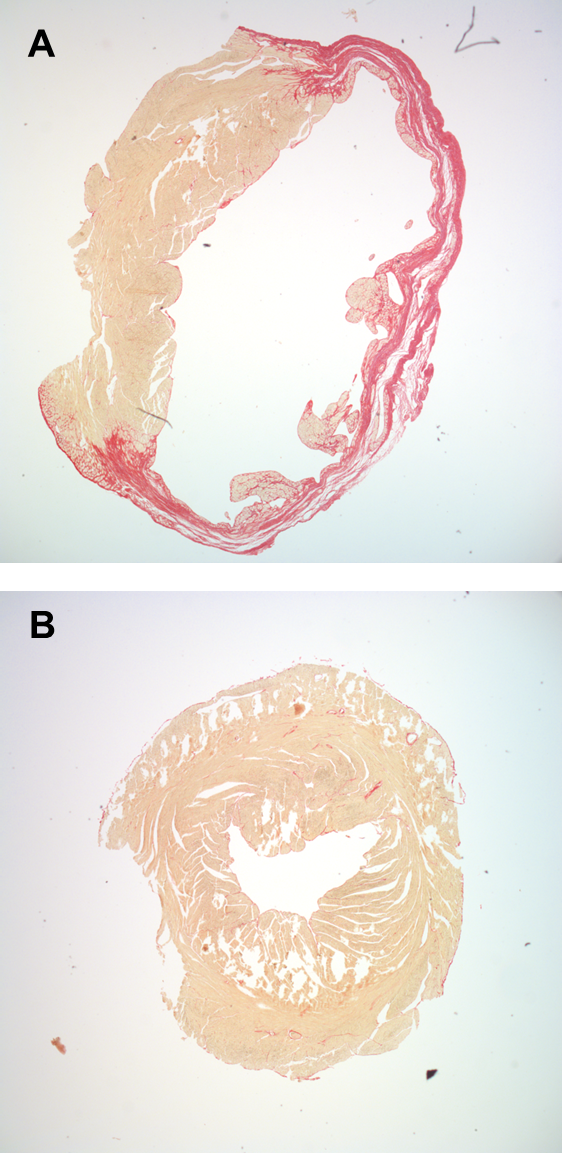

Supplement: Supplementary file 1 [file DataSheet1.DOCX]
